# Abstract of the Proceedings of the Buffalo Medical Association

**Published:** 1864-04

**Authors:** J. F. Miner

**Affiliations:** Secretary


ART. III.—*Abstract of the Proceedings of the Buffalo Medical Association.*

TUESDAY EVENING, March 1st, 1864.

Tbe President, Dr. Congar, in the Chair. The minutes of the last meeting read and accepted.

*Dr. Rochester* remarked upon the recurrence of Scarlet Fever, and related an instance where four children had the disease, an intelligent mother assuring him that they had all had it before. This, if true, he regarded as quite remarkable, yet not so strange since all had been exposed to the same influence. He also related an instance where scarlet fever, erysipelas and puerperal fever were all found at the same time, and in the same house; thought there was a well marked connection in these diseases. Had often attended cases of re-occurrence of scarlet fever; his own brother he had attended during two attacks, while he had not himself ever had the disease.

*Dr. Wetmore* had noticed the prevalence among the soldiers at Fort Porter, of acute pharangitis, the inflammation extending greatly in all directions. In some cases there was high fever; some appeared diphtheritic in character; these were more troublesome and dangerous than where there was no appearance of diphtheria. Had used the various astringents and caustics,—nit. silver, sul. zinc, tannin, persulphate of iron, etc. Would like to hear the experience of other members of the Association.

*Dr. Johnson* had been called to visit cases similar to those reported by Dr. Wetmore. Had observed that where there was the diphtheritic exudation the cases were more protracted and troublesome, and did not yield to treatment so readily. He had prescribed the usual remedies in such cases, and they had terminated favorably.

*Dr. Miner* remarked, that since they had passed the order of exercises for "voluntary communications," and no one had seen fit to offer anything under that head, he would present what he had to say under the present order of exercises, and call it an epidemic of *Fistula in Ano.* His attention had recently been attracted to the nature of this affection by having had several cases presented for operation, or for determining the propriety of operative treatment with view to its safety. Until within the past few years it was supposed to be unsafe to make radical cure of anal fistula, upon the ground that it acted as a sort of derivative, carrying off the impurities of the system, and protecting it from the encroachments of more serious disease. Idiopathic fistula, associated as it very often is with tubercular disease of the lung, was formerly regarded as almost a part of that disease, and certainly as exercising a great control over its progress and termination. That it is often found associated with consumption there can be no doubt, and in many instances should not be interfered with. In the late stages of consumption, when nothing is to be gained by the radical cure of any associate affection, and when all operative interference is dangerous, there can be no good argument for attempting its cure. Excluding this condition, the question of operation for radical cure of fistula, even when the general symptoms of tuberculosis are present, without as yet any local physical evidences of the disease, is an open one, and one which every practical surgeon is repeatedly called to consider and decide. It is very common to see patients with many of the general symptoms of consumption, who in reality have no disease of the lung; who suffer from fistula which has deranged the digestion, enfeebled the constitution, and induced a condition of debility and anæmia, not unlike that which attends tuberculosis, without of course the local evidences of consumption, though cough and expectoration are often present, induced by the condition of debility which has resulted from protracted suffering and profuse mucous and purulent discharge. It is almost unexceptional, that patients when cured of fistula, improve in flesh and strength; oftentimes this change is remarkable.—That people die after such operation is certain; that they sometimes die of consumption not long after, is quite probable; that they ever die in consequence of the cure of such disease is by no means established.

That fistula in ano induces the very disease for which it is often regarded as a cure, appears rational and philosophical. We see tuberculosis commence and progress when the system if reduced from any cause; and certainly a more direct and efficient agency cannot be conceived, than the constant purulent discharge, deranged digestion, pain and irritation which generally attends fistnla in ano. When this condition is produced by any other cause we do not hesitate to attempt its removal, and it will probably be difficult to show why attempt should not be made to remedy this condition of depression by curing the discharge, and by all the common remedies of stimulus and support, by which modern practitioners attempt to relieve or cure, all cases of debility from whatever causes they may spring. It is by no means a new view to take of this disease, and would not have been introduced this evening had there appeared anything more attractive. These considerations and many more connected with this subject, had forced themselves upon his attention in repeated instances during the last few weeks, and it was presented, since many members of the profession, still hold to the former doctrines of non-interference in this loathsome, and in most instances easily and uniformly curable disease, upon the ground that it is protective in its influence.

*Dr. Rochester* said that before dismissing the previous subject, he would call the attention of the members of the Association to the use of permanganate of potash as a topical application, in diphtheritic and other anginose disorders. Employed as a gargle two grains to an ounce of water; it entirely removed fœtor, and appeared also to allay irritation, and to induce a less morbid action. His experience in its employment was limited to five cases, and he mentioned it rather as an agent worthy of trial, than as a tested and approved remedy. As to the "epidemic" of fistula in ano, with which Dr. Miner's patients have been visited, and of which he has given us an account, he had nothing to say, but that gentleman's speculations as to the pathology and treatment of fistula in ano, especially in its relations to pulmonary tuberculosis, he could not but regard as erroneous, and indeed mischievous, if any one should be induced to adopt and act upon them. The question broached was not a new one, and Dr. R. was really sorry to see it revived in this Association, after a slumber of two years or thereabouts, for he was still conservative enough, or old fashioned enough to believe, that the accumulated experience of the profession for the past century—even if not given in a numerical or tabular form—was worthy of respect and consideration. That the voice of the profession was unanimous against operations for fistula in ano, in tubercular persons, no one would dispute, save that very small number who bring forward startling revolutions or novelties, more to make themselves conspicuous, than to really advance the interests of humanity or of the medical profession.—That fistula in ano is, as it were, a safety-valve to tubercular persons, was a fact as well established as it was possible, and scarcely any physician whose practice has extended over a period of ten or fifteen years, can fail to have had positive evidence to that effect. It is argued that as pulmonary tuberculosis results from exhaustion, debility and innutrition, that fistula in ano, by inducing these very conditions, develops tubercle, or hastens its progress when pre-existing. The premises, although true in the main, are by no means universally so, and the conclusion is entirely theoretical, and as before stated, the very opposite of the actual fact. Many instances might be cited in proof of this position, but it really seemed unnecessary to spend time in their rehearsal. The physician who examines a patient with fistula in ano, must be extremely careful in asserting that "the lungs are free from disorder," for the fistula not only acts as a derivative and safe-guard, but on the principle of "*ubi irritatio ibi fluxus,*" actually masks or silences the physical indications of pulmonary tubercle. By this assertion Dr. Rochester said that he wished to be distinctly understood to refer to the nice and delicate evidences of tubercular deposit, afforded by localized bronchitis, by roughened or prolonged expectoration, and by modifications of the pitch or note of respiration and vocalization. That when tubercle existed to such an extent as to produce dullness on percussion, or bronchophony, or to exclude air from a considerable portion of the lung—whether there was a fistula or not, it did not require a very delicate, practiced or attentive ear to determine the presence and the action, past or progressive of tubercle. Dr. Rochester did not claim that, in every instance anal fistula prevented the evolution or stayed the progress of pulmonary tuberculosis, and he would even admit that in some few and exceptional cases, that it would not only be proper but best to close the fistula, the precaution being always taken to precede the operation by the establishment of an issue in the arm, and subsequently to keep the same permanently in operation.

In conclusion Dr. Rochester said, that if he had spoken warmly, it was from his earnest conviction that a most fallacious theory had been broached, and that it was his duty to oppose it; that he disclaimed any personal reflection, and that he hoped his remarks would be received, as they were made, simply with reference to the welfare of suffering humanity.

*Dr. Miner* said that Dr. Rochester had replied to his remarks with more spirit than was common, when simple questions of science or fact were discussed. The accusation, that those who hold to and advocate the opposite from himself, have not the good of humanity or the profession at heart, but a desire to make themselves conspicuous, is so manifestly absurd, as to require no comment. "The accumulated experience of the profession for the past century," was indeed worthy of respect and consideration, and it was with the view of giving it this consideration and examination, that he had introduced this subject; a slumber in the Society of two years might well be broken. It is true that most physicians, of ten or fifteen years' practice, have seen fatal cases of consumption after operations for fistula; but it is by no means "established" that the cure of the fistula exercised any injurious influence. Every experienced surgeon has observed, after the cure of fistula, marked improvement in the general health and strength—has observed this even in cases with constitutional tendency to, and general appearance of, tuberculosis. They continue for years in better health and strength, but die sometimes years later, of consumption.—This is the "positive evidence," more common than any other, that fistula in ano acts as a "safety-valve in consumption." A physician must be very conservative and old-fashioned indeed not to discover the fallacy of the "accumulated experience of the past century in a matter upon which it is so impossible to obtain any like positive proof." The assertion made by Dr. Rochester, that fistula in ano "masks or silences the physical evidences of tuberculosis," is quite remarkable, and, even with his explanations and qualifications, amounts to the assertion that the earlier, nicer, and more delicate evidences of tubercular disease are not to be discovered when fistula exists, and therefore, though no palpable evidence of tubercule is present, operation should not be made for fear there may be undiscoverable consumption. Following this line of argument, ten or fifteen years later the patient dies of consumption, and it is set down as positive proof that the cure of fistula was the fatal mistake. It was distinctly stated, in introducing the subject, that fistula, in connection with tubercular disease, should many times be allowed to remain, as nothing could be gained by interference. If it acts as a safety valve, preventing the approach or retarding the progress of tuberculosis, it should certainly remain undisturbed; and substituting a similar, but less efficient protection, could hardly be regarded as justifiable. This idea, a part of that old and exploded system of counter-irritation, emetics, cathartics and starvation, has been re-printed in many standard works and brought down to us from a former generation. That such view should obtain support, when the nature and causes of both these diseases were much less understood, is not remarkable; but that it should continue to prevail, when all its fellow absurdities have become nearly extinct, is a matter of surprise and regret. He had often to combat this view in practice, and though he was not ambitious to have others adopt views similar to his own, he was glad of an opportunity to say that he believed it generally—almost always, desirable, in the treatment of any disease of debility, to relieve the system of all sources of irritation or exhaustion, and to restore it as speedily as possible to its accustomed vigor. Medicines, administered with that view, were useless and inoperative, unless the causes of debility and depression could be removed. Profuse suppuration or unnatural discharges from any cause were much more active in depressing than any known medication in raising the vital powers. This matter does not rest, as asserted, upon theoretical grounds alone. Cases, illustrative of the benefits of early operations in fistula, are by no means wanting, and when we are told that years are necessary to complete our record, we can but think that half the same period would have sufficed had no operation been made.

*Dr. Rochester* replied that he had no desire to prolong the discussion. Whether his remarks deserved the imputation cast upon them, or whether they would bear the interpretation given them, he preferred to leave to others to determine. On one point however, there was evidently a very material misapprehension. He had spoken of a professional experience of ten or fifteen years, as necessary for proper medical observation, without reference to that time, as applied to the course or progress of tubercle, in any given case. The history of the diseased condition, in question, in most instances, is something like this: A person has a cough, has had perhaps hæmoptysis, physical exploration determines to a greater or less extent the presence of pulmonary tubercle. Fistula in ano sets in. The cough ceases; years pass by, (in one instance eighteen.) The general health, if not robust, is fair. The patient becomes tired of the local annoyance, is operated upon, and is happy for a brief period, but in a few months the old cough returns, and in from one to three years death ensues from phthisis pulmonalis.

On motion of *Dr. Wyckoff* it was

*Voted* that Dr. Brown, House Physician and Surgeon to the Buffalo General Hospital, be invited to participate in the proceedings of the Association until time was afforded to complete his membership in the County Society.

Dr. Ayer was proposed for membership.

*Voted* to adjourn to the first Tuesday evening in April.

J. F. Miner, Secretary.

---

Appointments Confirmed.—The Senate has confirmed the appointment of Medical Inspector Joseph K. Barnes, U. S. A., to be Medical Inspector General, U. S. A., with the rank of Colonel.

